# Large Bandgap Shrinkage from Doping and Dielectric Interface in Semiconducting Carbon Nanotubes

**DOI:** 10.1038/srep28520

**Published:** 2016-06-24

**Authors:** Everett Comfort, Ji Ung Lee

**Affiliations:** 1 Colleges of Nanoscale Science and Engineering, SUNY-Polytechnic Institute, Albany, NY 12203, USA.

## Abstract

The bandgap of a semiconductor is one of its most important electronic properties. It is often considered to be a fixed property of the semiconductor. As the dimensions of semiconductors reduce, however, many-body effects become dominant. Here, we show that doping and dielectric, two critical features of semiconductor device manufacturing, can dramatically shrink (renormalize) the bandgap. We demonstrate this in quasi-one-dimensional semiconducting carbon nanotubes. Specifically, we use a four-gated device, configured as a *p-n* diode, to investigate the fundamental electronic structure of individual, partially supported nanotubes of varying diameter. The four-gated construction allows us to combine both electrical and optical spectroscopic techniques to measure the bandgap over a wide doping range.

The bandgap is one of the most important electronic properties of a semiconductor. For example, the bandgap determines the emission wavelength of LEDs and Lasers. In transistors, the bandgap determines the on/off state which is important for power dissipation^1^. Semiconducting single-walled carbon nanotubes (SWNTs) are promising materials for optoelectronic and electronic applications^2^,^3^,^4^. They are quasi-one-dimensional (1-D) materials that are characterized by (n,m) chiral indices, which define their structure and diameter. Certain (n,m) indices give rise to a semiconducting behavior. SWNTs exhibit reduced carrier scattering^5^, enhanced optical absorption^6^, and bandgaps tunable with diameter^7^. Here, we discuss the challenges associated with measuring the bandgaps of intrinsic SWNTs and the approach we have developed using a four-gated *p-n* diode to measure them. Previously, either the electronic characterization or the optical spectroscopic techniques were used to measure the properties of individual SWNTs, without the ability to control doping. Although the importance of many-body effects was recognized early on from the measurement of excitonic levels^8^, an experimental study on its impact on the electronic (quasiparticle) bandgap has received little attention.

In our work, we use a four-gated device to measure both the electronic and optical properties of partially supported SWNTs. We perform these measurements on individual nanotubes over a wide doping range. We show that the bandgap is dominated by many-body effects that shrink its value dramatically. In the low-doing limit, we show that dielectric screening from the SiO_2_ substrate renormalizes the bandgap. With doping using gating techniques, the bandgap shrinks further due to enhanced electron-electron interaction from the 1-D confinement. We show that both effects are large and demonstrate their diameter dependence for the first time. These features are expected to be universal for all low-dimensional systems where large excitonic effects occur^9^,^10^.

The measurement of the intrinsic bandgaps of semiconducting SWNTs has been elusive due the inherent difficulty in measuring them in 1-D semiconductors^11^. As Fig. 1(a) shows, the electronic structure of SWNTs is characterized by subbands. Each subband gives rise to a van Hove singularity (vHs) in the density of states. Figure 1(a-ii) shows the first two vHs for the two lowest subbands. While an optical absorption technique can be used to measure the band-to-band transition in bulk semiconductors, it is difficult to measure the interband transition in the linear optical regime of SWNTs. The difficulty arises due to the weak oscillator strength of the band-to-band transition compared to the excitonic transition. We illustrate this in Fig. 1(a-iii) for the first subband. It shows the prominent excitonic transition (labeled *E*_*11*_) compared to the band-to-band transition *E*_*g1*_. The exciton binding energy *E*_*B*_, the separation between *E*_*11*_ and *E*_*g1*_, is predicted to be as large as 1 eV^12^,^13^. In essence, the excitonic transitions steal away the oscillator strength from the vHs, making them the dominant peaks. Therefore in an undoped SWNT optical transitions, either by absorption or emission, are unable to measure the intrinsic bandgap reliably. Alternatively, using scanning tunneling spectroscopy (STS) to measure the bandgap requires that the nanotube be in intimate contact with a conducting substrate, typically a metal. The metal substrate, however, can lead to charge transfer and screening of Coulomb interaction. As we show below, both doping and the dielectric environment have a dramatic effect in renormalizing the bandgap. In the early studies of STS^14^,^15^, a diameter dependence that coincided with the one from a single-particle picture was observed. Here, we examine this value in the context of both the photophysics and electronic properties of SWNTs for different diameter nanotubes and challenge its ubiquitous use to characterize SWNTs. We focus specifically on the low-doping characteristics, which is permitted by our special device construction.

Figure 1(b) provides a summary of both the electronic and optical properties of different (n,m) SWNTs^7^ as a function of diameter (*d*). Circles are the two lowest optical excitonic transitions *E*_*11*_ and *E*_*22*_for the first two subbands. Filled circles (in green) are predicted values (also known as the Kataura plot)^16^, and open circles are measured data^17^. The open red circles are data from our diodes. Figure S3 shows an example of excitonic transitions in the photocurrent spectrum of a SWNT diode. It is used to assign the diameter accurately using the Kataura plot. The photocurrent spectrum originates from the region between the inner gates, to be described below, where the nanotube is suspended. *Ab initio* calculations predict that the first bandgap *E*_*g1*_is situated about midway between *E*_*11*_ and *E*_*22*_ levels^12^,^13^,^18^. In Fig. 1(b), this region is shown as a band because different theories predict slightly different values. *E*_*g1*_ would be measured if the SWNTs were in a vacuum, which is difficult to realize in practice since SWNTs are usually supported by a dielectric environment or can be inadvertently doped. As a reference, we show the bandgap from a single-particle tight-binding (TB) calculation. The TB bandgap predicts a value *E*_*g*_ = 2*γ*_*o*_*a*_*cc*_/*d*, where *γ*_*o*_ is the nearest neighbor hopping integral and *a*_*cc*_ is the carbon-carbon distance (0.14 nm). Taking *γ*_*o*_ = 2.90 eV^19^ yields a TB bandgap of *0.82 eV/d*(*nm*), where *d* is in nm. It is clear from this figure that one cannot reconcile the position of the TB bandgap since calculations predict that the true bandgap should be above the *E*_*11*_ levels by *E*_*B*_, i.e. at *E*_*g1*_. The large difference between the two bandgap positions shown in Fig. 1(b) suggests that we have been guided by an incomplete understanding of the electronic structure of SWNTs. Here, we reconcile the different between previous measurements and theory, and provide new insights on bandgap renormalization (BGR) based on our four-gated diodes.

## Results

Our approach uses a four-gated device to create *p-n* diodes along individual nanotubes. These diodes follow Shockley’s celebrated ideal diode equation^20^, which allows us to measure the intrinsic properties of nanotubes, including the bandgap. Specifically, we measure the doping-dependent bandgap of nanotubes over a wide range, which has not been possible until the development of the four-gated diode. The doping induced bandgap renormalization is a many-body effect that is enhanced in 1-D systems^10^,^21^. Using these diodes, we show that the bandgap is a strong function of doping. In the low-doping regime, the SWNT is characterized by a doping-independent renormalization from dielectric screening.

In the absence of doping techniques, one can use two buried gates (see Fig. S1(a) in supplementary information) that are biased with opposite voltages to create a *p-n* junction diode. We have previously reported on the formation of SWNT *p-n* junction diodes using two gates and their optical properties^22^,^23^. This technique has been used to create *p-n* junctions in a wide range of materials, including graphene^24^ and 2-D transition metal dichalcogenides^25^,^26^,^27^. One limitation of using only two gates to create a *p-n* diode is that the bias on the gates has to be large to observe the diode behavior. The large voltage is needed because the contacts to these materials often form Schottky junctions, and the large voltage reduces the contact resistance, which can mask the diode behavior. Therefore, the two-gated diode construction fundamentally constrains the device to operate only at high doping regimes when the bandgap has renormalized significantly.

Here, we extend the exploration of SWNT diode properties by using a four-gated construction, as shown in Fig. 2(a–c), to examine the bandgap in the low-doping limit. The two outer gates (***V***_***g,out***_) are biased with large voltages, just as in the two-gated devices, and make the Schottky tunnel barriers small. The two inner gates (***V***_***g,in***_), on the other hand, can be biased to achieve any doping level to study the effects of BGR. Figure 2(b) shows a scanning electron microscope image of a four-gated device, and Fig. 2(c) is an example of how we bias the gates. A detailed fabrication process is described in Methods. Briefly, the gate dielectric is 100 nm thick SiO_2_ and we create trenches between the gates to create nearly ideal diodes^28^. The trench spacing between the inner gates is wider (between 1–1.5 μm) than that between the outer and inner gates (0.1–0.3 μm), as shown in Fig. 2(b).

Figure 2(d) shows a typical diode current-voltage (I–V) characteristics from a four-gated device for different ***V***_***g,in***_. To construct a set of *I–V* curves, we fix ***V***_***g,out***_ at a high bias (+/−12 V in this case) and vary ***V***_***g,in***_ (the bias in the inset represents +/−1 V to +/−16 V on ***V***_***g,in***_ gates). The characteristics at low ***V***_***g,in***_are in sharp contrast to those we measure in a two-gated device, as we show in Fig. S1(b). There, we clearly observe the limitations of using only two gates. As ***V***_***g,in***_is reduced, the diode characteristics become masked by the Schottky contacts, and the trend of decreasing *I*_0_ with decreasing ***V***_***g,in***_seen at other gate voltages is broken at ***V***_***g,in***_ = +/−1 V. This limitation is not observed in the four-gated device in Fig. 2(d). We note that *I*_0_ is independent of ***V***_***g,out***_, as we show in Fig. S2 by varying ***V***_***g,out***_ at a fixed ***V***_***g,in***_ = +/−8 V. We observed similar behavior for other ***V***_***g,in***_values, indicating that minority carriers near the *p-n* junction determine the diode characteristics. To characterize our devices, we fit the diode I–V characteristics to the diode equation *I* = *I*_0_(*exp*(*qV/nKT*)*−*1), where *I*_0_ is the leakage current, *q* the elementary charge, *V* the applied voltage, *K* the Boltzmann constant, and *T* the temperature. *n* is a measure of the ideality of the diode, and these diodes can be fit with n ~ 1.1 (See Fig. S1(c))^28^, close to the theoretical limit of 1. In an ideal diode, *I*_0_ parameterizes both the recombination (V > 0) and generation (V < 0) of minority carriers, which are generated over the bandgap energy. Here, we use *I*_0_ to measure the bandgap properties as we vary ***V***_***g,in***_, focusing on the low-doping characteristics to minimize the doping induced BGR. In the inset of Fig. 2(d), we plot the extracted *I*_0_ as a function of ***V***_***g,in***_. The large change in *I*_0_ seen in Fig. 2(d) can be related directly to the quasiparticle bandgap energy *E*_*g*_ by measuring the activation energy *E*_*a*_ of *I*_0_, as we discuss below.

In Fig. 2(d) as ***V***_***g,in***_ increases, we observe an overall upward shift in the I–V curves, i.e. *I*_0_ increases as the doping increases while maintaining the nearly ideal diode behavior. This trend is completely opposite to the general theory and observation in bulk diodes^29^. The increase in *I*_0_ with doping is a direct result of a significant doping-induced BGR, since *I*_*0*_ ~ *exp*(−*E*_*g*_/*KT*)^1^. In our model, *I*_0_ originates from the diffusion of minority carriers in the doped regions near the junction, just as in bulk diodes. In the absence of or with weak doping-induced BGR, an increase in doping results in fewer minority carriers, which decreases *I*_0_1. This is seen in bulk *p-n* diodes where doping induced bandgap shrinkage is small^29^. It is an order of magnitude less than in a SWNT for an equivalent doping density^30^,^31^,^32^. In SWNTs, however, we observe the opposite behavior to bulk diodes because of the larger change in the bandgap than the opposing change in *E*_*F*_ with increased doping, discussed below in our conclusion. As discussed theoretically, the doping-induced BGR can reduce the bandgap significantly in SWNTs^21^. To account for the significant increase in *I*_0_ with doping, a BGR of more than half the intrinsic bandgap is needed at the highest doping density we have examined, consistent with results from ref. 21. A similarly large BGR is also predicted in surface-gated and modulation-doped semiconductor quantum wires^33^,^34^. The similarity between results from other 1-D systems with those of SWNTs highlights the significant role of many-body effects in 1-D semiconductors.

We substantiate the large BGR indicated in Fig. 2(d) by measuring the activation energy *E*_*a*_ of *I*_0_ for different ***V***_***g,in***_ values. In Fig. S4, we show how we extracted *E*_*a*_ for the device in Fig. 2(d). To measure *E*_*a*_, we plot the I–V curve at each ***V***_***g,in***_ and temperatures T, which is varied from 300 K to 330 K. Next, each I–V curve is fit to the diode equation to determine *I*_0_. Finally, *E*_*a*_ is determined from the plot of *I*_0_ vs *T* using an Arrhenius relation. We observe a large change in *I*_0_ with both temperature and ***V***_***g,in***_, which allows us to make a precise determination of *E*_*a*_ for each ***V***_***g,in***_ value. In Fig. 3, we plot *I*_0_ and *E*_*a*_ for several diodes at different doping levels. The variation in *I*_0_ seen in different devices is due to different bandgap nanotubes. We note that *E*_*a*_ is largely independent of the reverse bias voltage despite the small increase in *I*_0_ with reverse bias. We also include results from two-gated devices as a reference in Fig. 3(a–c). The lowest doping density that we are able to achieve while being able to observe the diode behavior for the two-gated devices is higher than that for the four-gated device. Nevertheless, there is good agreement between the two-gated and the four-gated devices.

To convert the bias ***V***_***g,in***_ to doping density, we use the geometrical capacitance per length calculated using 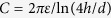, which we determined from a finite-element calculations (because of the large dielectric thickness we use, quantum capacitance can be ignore). Here, ε is the dielectric permittivity of SiO_2_, *h* is the SiO_2_ thickness (100 nm), and *d* is the diameter of the SWNT. In using this capacitance for all voltages, we overestimate the true carrier density at low bias since the nanotube is effectively a dielectric when the doping in the nanotube is below the degenerate doping level. This error is not important for our discussion since we focus on the low-doped regime where the characteristics saturate.

The data in Fig. 3 shows an inverse correlation between *I*_0_ and *E*_*a*_: the larger the *I*_0_, the lower the *E*_*a*_. This applies to other nanotubes and over the wide doping range we have examined. The results support the conclusion that *I*_0_ originates from the generation of minority carriers, which are activated over the bandgap energy. When the doping is low and the Fermi level *E*_*F*_ is within the bandgap, *E*_*a*_ is equal to the bandgap energy, independent of how much the bandgap has renormalized. At large doping densities, however, when the Fermi level *E*_*F*_ has moved into a band, *E*_*a*_ is no longer equal to the bandgap energy, as we show in Fig. 4(c). Instead, *E*_*a*_ is larger than the bandgap energy by the separation between the Fermi level *E*_*F*_ and the band edge. This is because now the minority carriers have to be activated also over the filled level. Below, we focus on the characteristics at low-doping where doping induced BGR is expected to be small.

The most important feature in Fig. 3(a–c) is that both 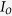 and *E*_*a*_ saturate at low doping densities. The saturation is expected since doping-induced BGR becomes smaller with decreased doping. In Fig. S5, we show that no additional information can be gained by reducing ***V***_***g,in***_ below +/−1 V, as the saturation in both 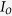 and *E*_*a*_ persists down to ***V***_***g,in***_ = +/−0.1 V, the lowest voltage we have tested. The equivalent carrier density at +/−0.1 V is less than 10^5^ cm^−1^, a low doping concentration that still permits the formation of a well-defined *p-n* junction. Figure 3(d) summarizes the results, where we focus only on the four-gated devices that allow us to measure the properties at low-doping values. We plot both *E*_*a*_, for different *V*_*g,in*_ values, and *E*_*11*_ as a function of *d*. It shows that the highest *E*_*a*_ we extract is well below the *E*_*11*_ values, suggesting that the bandgap is still renormalized significantly even though the doping is low. The dotted curve in 3(d) is a fit to the data at ***V***_***g,in***_ = +/−1 V. In Fig. S6, we provide fits to a 1/d relation for both two- and four-gated devices over an expanded doping range. We arrive at the conclusion that in the limit of low doping, the lowest quasiparticle bandgap is given by *E*_*g*_ = *0.81* *eV*/*d*(*nm*).

Figure 3(d) embodies the central results of this paper and leads to the surprising conclusion that *E*_*a*_ extrapolates to the single particle bandgap in the limit of low-doping. We believe that this is not coincidental and merits further investigation. The experimental determination of the bandgap dependence with diameter using STS on individual SWNTs showed similar results^14^,^15^. There, the nanotubes were placed on Au, which is a very different substrate than the SiO_2_ that we use. These investigators measured *E*_*g*_ of *0.69* *eV*/*d*(*nm*) and *0.76* *eV*/*d*(*nm*), which are somewhat lower than the bandgap value we measure. There, charge transfer doping would have been inevitable between two materials (Au substrate and SWNTs) with different workfunction^35^. In another study of STS on SWNTs in a bundle^36^, a BGR was observed with nanotube-substrate separation. There, however, one cannot differentiate between doping and dielectric screening as the cause of the renormalization. In our case, it appears that SWNTs are characterized by a BGR that is doping independent.

## Conclusions

**Figure 1:**
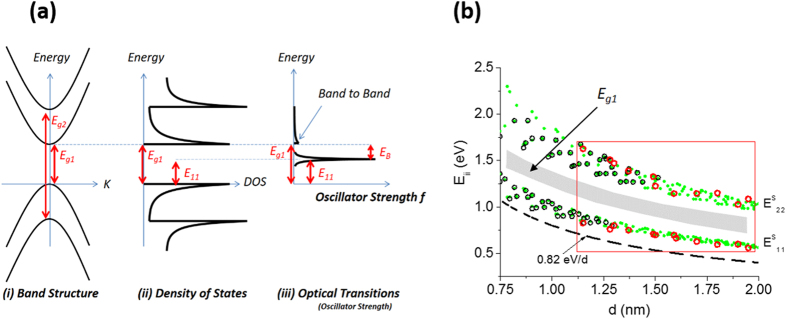
(**a**) Electronic and optical properties of a SWNT: (i) The electronic dispersion relation of a SWNT. Since a SWNT is a quasi 1-D semiconductor, subbands are formed with bandgaps *E*_*g1*_*, E*_*g2*_ etc. (ii) Associated with each subband is a density of states (DOS) that exhibit a singularity called the van Hove singularity. (iii) Focusing on the first subband, the optical transition is dominated by the lowest optically active excitonic transition *E*_*11*_ (*f* is oscillator strength). In essence, the excitons steal away the oscillator strength from the continuum (the bandgap). Therefore, it is difficult to measure the bandgap by using optical absorption or emission techniques. (**b**) Excitonic levels from calculations (closed circles–green) and measurements (open circles) for different (n,m) chirality nanotubes. The values within the rectangle (red circles) are measured from our p-n diodes formed along individual SWNTs. The shaded area is the expected region for the intrinsic band gap for the first subband.

**Figure 2:**
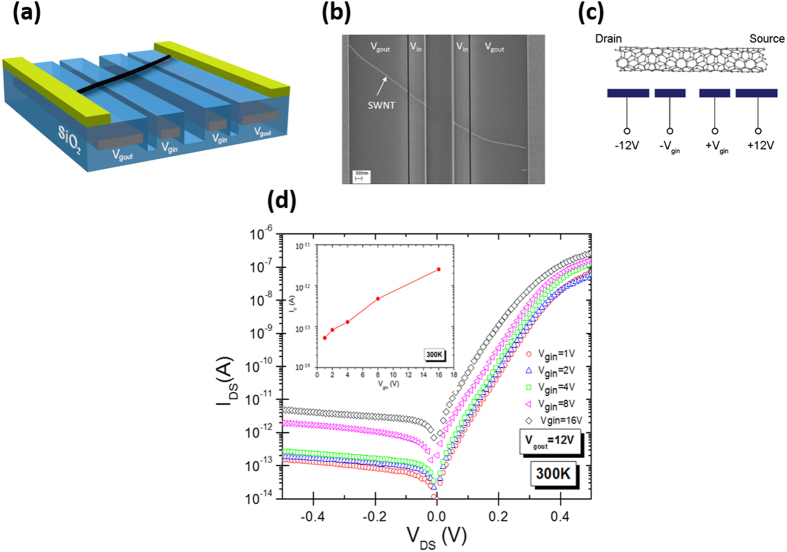
4-gated SWNT p-n diodes formed along individual nanotubes. (**a**) Show the four-gated diode construction and (**b**) is an SEM image. (**c**) A typical bias configuration to achieve low Schottky contact resistance while allowing arbitrarily low doping levels with *V*_*g,in*_. (**d**) The current-voltage characteristic of a four-gated diode as a function of *V*_*g,in*_. *V*_*g,out*_ is fixed at +/−12 V while *V*_*g,in*_ is varied according to the values in the inset. The increase in Io with increasing Vgin is a direct result of doping-induced bandgap shrinkage. The inset in (**b**) shows the extracted *I*_*o*_ using the diode equation.

**Figure 3:**
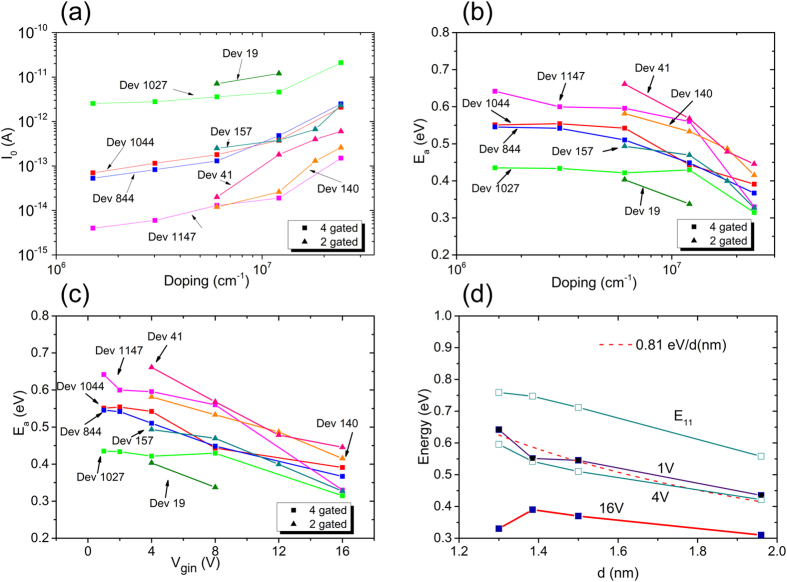
(**a–c**) We plot *I*_0_ and *E*_*a*_ for different SWNT diodes as a function of *V*_*g,in*_. The bias on the gates (shown in (**c**)) is converted to carrier density using the geometrical capacitance between the nanotube and the gate. (**d**) We plot the measured *E*_*a*_ and *E*_*11*_ for different *V*_*g,in*_ values as a function of diameter. The diameter is assigned to each nanotube based on the Kataura plot shown in Fig. 1(b). At *V*_*g,in*_ = +/−1 V, *E*_*a*_ *s*aturates to a value of 0.81 eV/d(nm), close to the TB bandgap.

**Figure 4:**
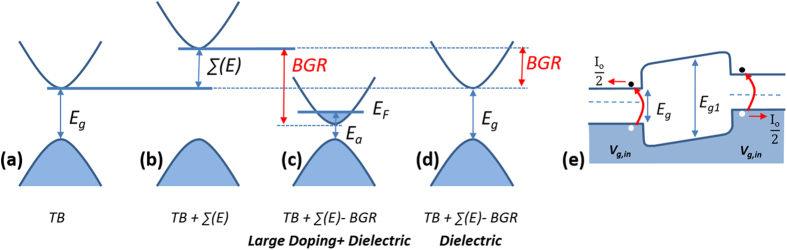
Electronic structure from doping and dielectric environment, and the resulting band diagram. (**a**) The TB electronic structure. (**b**) Electron-electron interaction adds a self-energy correction ∑(*E*), which is estimated to nearly double the TB band gap. From (**b**), the bandgap can be renormalized due doping (**c**) and from the dielectric environment (**d**). In the case of heavy doping (**c**), the activation energy for electron-hole generation is larger than the actual band gap. Under nondegenerate doping, the bandgap can be measured directly from Io in Eq. (1). (**e**) The band diagram of a p-n diode at low doping and under a small reverse bias. It shows that carriers that give rise to *I*_0_ are generated in the regions above *V*_*g,in*_ with bandgaps that are renormalized due to dielectric screening.

In Fig. 4, we summarize and reconcile our results with those from calculations. In a single-electron approximation (Fig. 4a), for example using a TB calculation, one arrives at a bandgap that scales as *0.82* *eV*/*d*(*nm*), as noted previously. The inclusion of electron-electron interaction to the single-particle picture adds a self-energy correction ∑(*E*), which opens the bandgap dramatically, almost doubling the TB bandgap (Figs 1b and 4b). When doped, due to exchange and correlation effects, the bandgap reduces (Fig. 4c). The bandgap reduction is the origin of the low *E*_*a*_ we measure at the highest doping density in Fig. 3. At the highest doping we examined, we estimate the band gap is about 0.3 eV below the measured *E*_*a*_ based on the calculated position of *E*_*F*_. In the low-doping limit, the fact that *E*_*a*_ remains well below the *E*_*11*_ values suggests that a doping-independent BGR dominates the SWNT characteristics.

A recent analysis of SWNTs on substrates also predicts a large BGR in the absence of doping^37^,^38^. These authors show that the dielectric screening from a substrate reduces the self-energy correction, making the quasiparticle bandgap smaller than that of a SWNT in vacuum (Fig. 4d). In Fig. 4(e) we show the resulting band diagram that incorporates BGR from the dielectric screening in the low-doping limit. It explains the saturation in *E*_*a*_ that we measure in the limit of low-doping. The regions marked ***V***_***g,in***_ have a significantly reduced bandgap that allows electron-hole generation with *E*_*a*_that is lower than *E*_*11*_. In contrast, the region between ***V***_***g,in***_ should take on the full bandgap shown in Fig. 4(b). Theory predicts that the conduction and valence bands renormalize equally^38^, resulting in the band diagram shown in Fig. 4(e). Lastly, the similarity between the results from STS and our measurement should be explored further. It suggests a hitherto unexplored mechanism that seems to result in near exact cancelation of ∑(*E*), independent of the dielectric environment of the supporting substrate. One potential area of exploration is to investigate the magnitude of BGR that combines the effects from both doping density and dielectric environment, particularly between those of SiO_2_ and metal.

## Methods

We fabricated the 4-gated devices at SUNY Poly’s 300 mm silicon wafer fabrication facility. The starting substrate is a 300 mm silicon wafer. We first grow 1 μm of SiO_2_ to create the isolation for the buried gates. Next, we deposit 100 nm of heavily doped n-type poly-crystalline silicon (poly-Si) film which will form the gates. We are able to achieve a doping density of ~10^19^–10^20^ cm^−3^, which makes the poly-Si highly conductive. Using photolithography and reactive ion etching (RIE), we pattern the gates with spacing that vary from 0.3 μm to 2 μm. After patterning the gates, 300 nm of SiO_2_ is deposited using a plasma-enhanced chemical vapor deposition (PECVD) process with tetra-ethyl-ortho-silicate (TEOS) and ozone precursors. To remove the resulting topography, we use chemical mechanical planarization (CMP) process. After CMP, the surface is planarized, and a thin layer of SiO_2_, about 10–20 nm thick, remains above the poly-silicon. Next, we deposit a second SiO_2_ layer to achieve a final gate oxide thickness of 100 nm. Next, bondpads are etched into the SiO_2_ to make electrical contacts to the poly-silicon. This is followed by depositing and etching 50 nm of TiN or Mo metal to define the electrical pads. Both were selected for their ability to make good electrical contacts to SWNT and withstand the high SWNT growth temperatures. To suspend the SWNT in the junction region, we etch trenches between adjacent split gates. This is accomplished using photolithography and etching into the SiO_2_, to a depth of ~450 nm. We suspend the nanotube in this manner as this has been shown to result in nearly ideal diode behavior compared to completely supported SWNTs. Finally, we define the location for SWNT catalyst by patterning PMMA over the metal contacts. The catalyst solution consists of 20 mg of anhydrous iron nitrate dissolved in 30 mL of methanol and is mixed with 30 mg of fumed alumina nanoparticles^39^. After spin coating, the PMMA is lifted off in acetone, leaving lithographically patterned SWNT catalyst regions. In each of these catalyst regions, the alumina nanoparticles act as a support for the iron nitrate, which forms small nanoparticles that will catalyze SWNT growth. Finally, the die is placed into a hot wall quartz tube furnace, and carbon nanotubes are grown by flowing methane in a chemical vapor deposition process. We probe the devices and select ones with semiconducting nanotubes. All measurements are conducted in a high vacuum system at T = 300 K, unless stated otherwise. In addition, we have confirmed in SEM and with excitonic spectra that only one nanotube bridged the contacts for the devices that we report. For measuring the activation energy E_a_, we varied T from 300 K to 330 K, which was sufficient to measure *E*_*a*_ accurately without affecting the device characteristics that might obscure its measurement.

## Additional Information

**How to cite this article**: Comfort, E. and Lee, J. U. Large Bandgap Shrinkage from Doping and Dielectric Interface in Semiconducting Carbon Nanotubes. *Sci. Rep.* **6**, 28520; doi: 10.1038/srep28520 (2016).

## Supplementary Material

Supplementary Information
