# Supplementary material for: Large Bandgap Shrinkage from Doping and Dielectric Interface in Semiconducting Carbon Nanotubes
Source: Sci Rep. 2016 Jun 24;6:28520. doi: 10.1038/srep28520 (PMC4919786; doi:10.1038/srep28520)
Supplement: Supplementary Information [file srep28520-s1.pdf]

Large Bandgap Shrinkage from Doping and Dielectric Interface in Semiconducting Carbon Nanotubes

Authors: Everett Comfort and Ji Ung Lee

Supplementary Materials

Two-gated SWNT p-n diode:

A two-gated device construction (Fig. S1(a)) is typically adopted in the fabrication of p-n devices with no known doping techniques. However, this limits the diode to operate only at large doping densities. Its fabrication is identical to the four-gated device described in the main text.

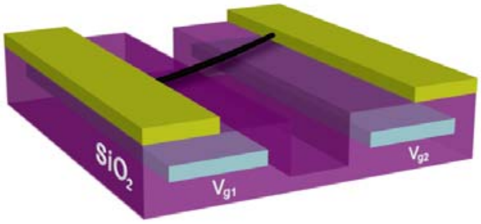

(a)

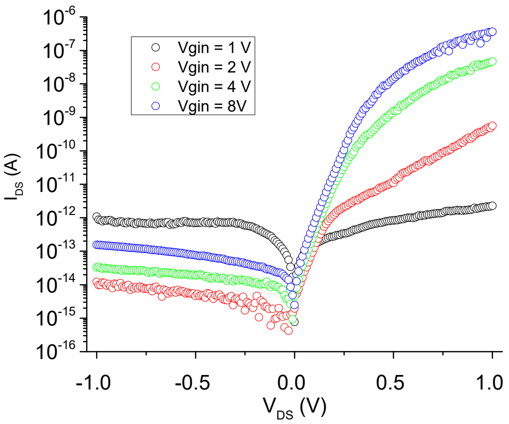

(b)

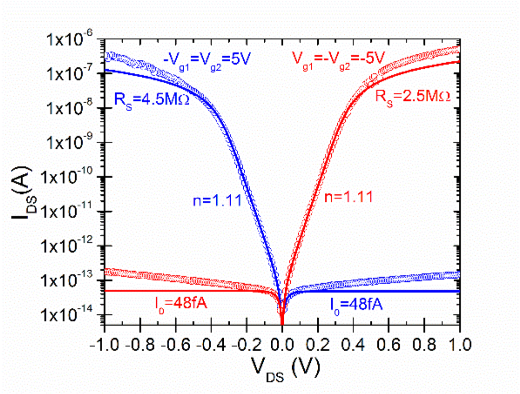

(c)

Fig. S1: (a) In the two-gated device construction, the diode properties can be observed only when the bias on the gates is large, when the bandgap has renormalized (BGR) significantly. (b) This is seen in the I-V curve in at Vgin=+/- 1V, which no longer follows the trend seen at higher bias because the characteristics are limited by the Schottky contact resistance. (c) Fit to the diode equation with n=1.11 and a series resistance for the two diode polarities we can achieve.

Dependence of Io on $V_{g,out}$ and $V_{g,in}$:

The I-V characteristics of a four-gated device showing a large change with $V_{g,in}$ at a fixed $V_{g,out}$ (Fig. S2-(a)), but showing little change with $V_{g,out}$ at a fixed $V_{g,in}$ (Fig. S2(b)). This shows that the diode characteristics are dominated by the p-n junction region from the inner doped regions.

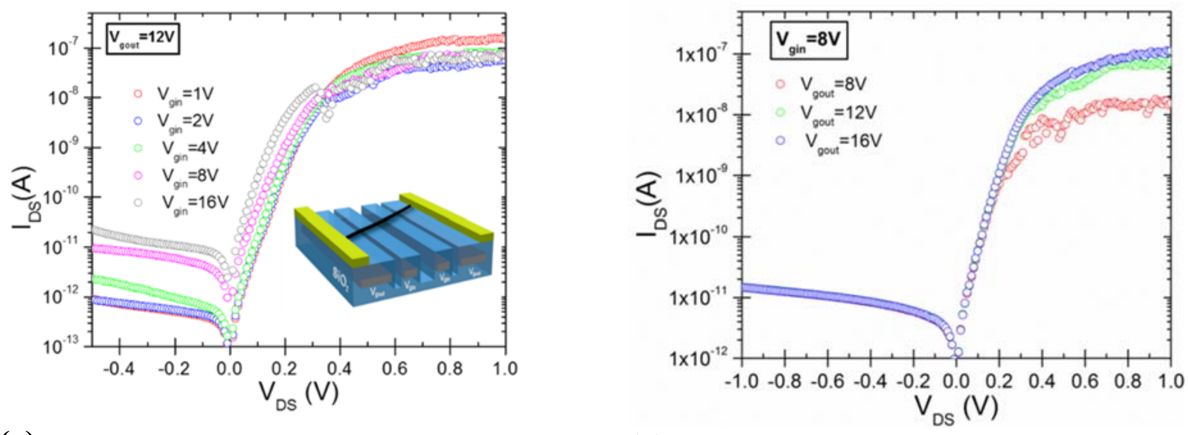

(a) **(b)**

Fig. S2: I-V characteristics of a four-gated diode as a function of (a) $V_{g,in}$ for a fixed $V_{g,out}$ and (b) $V_{g,out}$ for a fixed $V_{g,in}$. The bias in the insets represents +/- polarities. The large increase in Io with $V_{g,in}$ is due to a significant change in the bandgap with doping, as discussed in the main text.

Photocurrent Spectra for a single SWNT configured as a p-n diode:

Fig.S3 shows the photocurrent spectra taken on a single SWNT configured as a p-n diode. We have shown previously that the spectra is independent of the gate bias but is quenched when the region between the inner gates is doped using the global Si back gate(*1*). This shows that the spectrum is from the region between the gates where there is little doping. When the middle region is doped, screening from the free carriers reduce the excitonic effects.

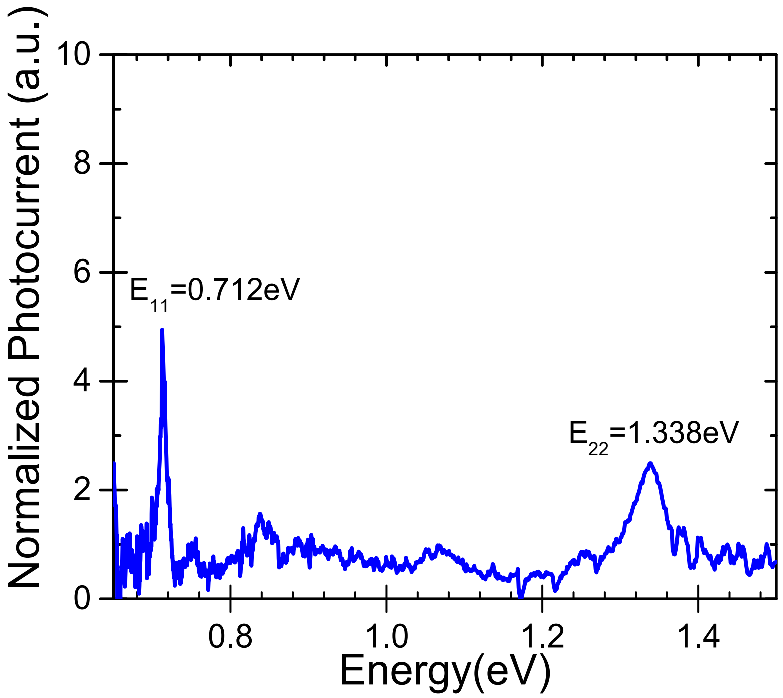

Fig. S3: Typical photocurrent spectra of a SWNT p-n diode taken at 300K. The spectra is independent of the bias on the gates, suggesting that the excitons are generated in the region between the inner gates where doping is low. E11 and E22 are the two lowest optically active excitons for the two subbands we show in Fig. 1(a).

Activation energy measurement:

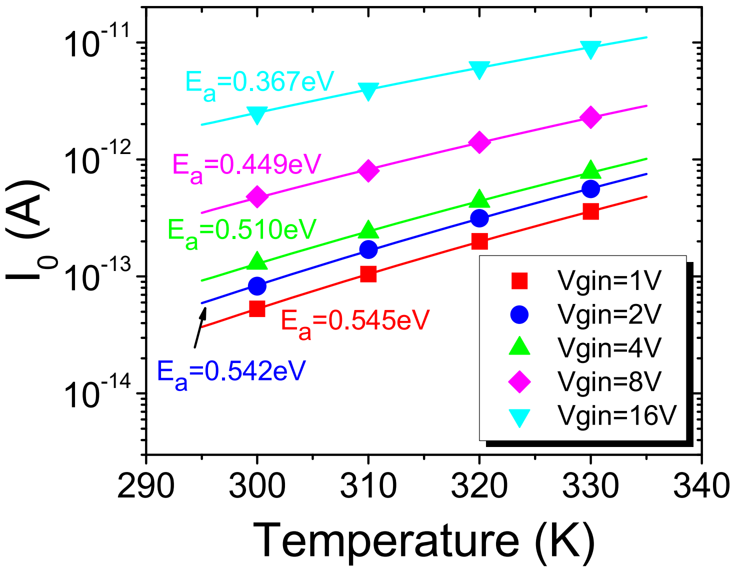

Fig. S4: We measure the activation at each $V_{g,in}$ by varying the temperature (T) from 300K to 330K in 10 degrees increment. At each temperature and bias, we measure the I-V characteristics and fit it to the diode equation described and text. The extracted Io vs T is plotted here. Next, we extract the activation energy Ea by fitting the plot at each $V_{g,in}$ to the Arrhenius relation $I_o \sim \exp(-E_a/KT)$.

I-V characteristics at Vg,in down to +/- 0.1

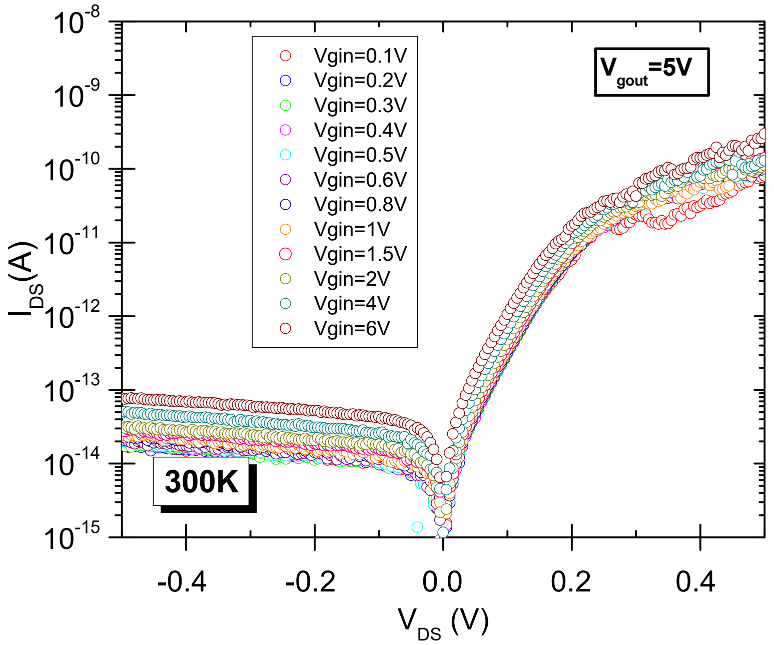

(a)

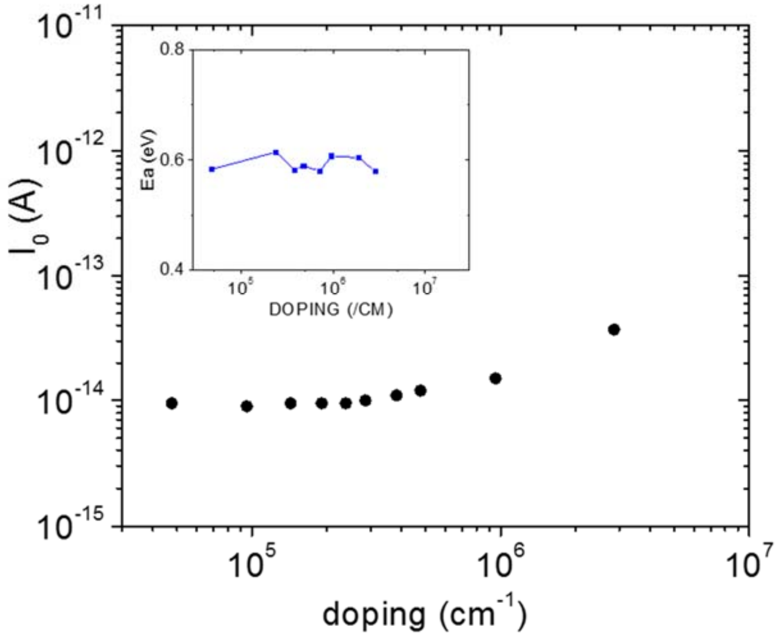

(b)

Fig. S5: (a) I-V characteristics down to Vg,in=+/- 0.1 V, which corresponds to less than 10^5 cm^{-1} carrier density. The Io and Ea for these curves are shown in (b).

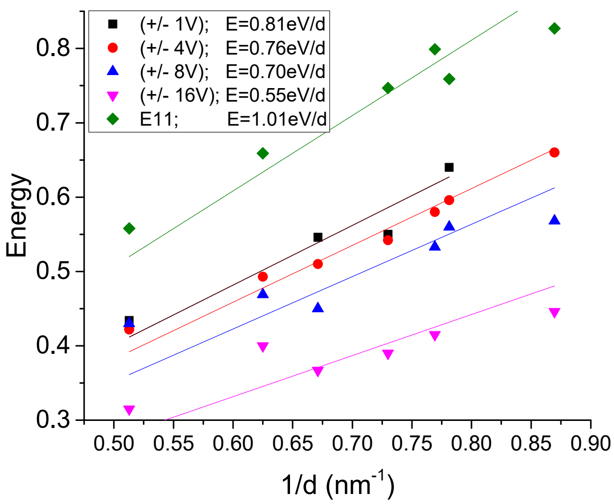

Fig. S6: Plot of E_{11} and of E_a for different diameter SWNT p-n diode as a function of $V_{g,in}$, from both two- and four-gated devices. The lines are linear fits to a 1/d relation, which are shown in the inset.

References:

1. A. Malapanis, V. Perebeinos, D. P. Sinha, E. Comfort, J. U. Lee, Quantum Efficiency and Capture Cross Section of First and Second Excitonic Transitions of Single-Walled Carbon Nanotubes Measured through Photoconductivity. *Nano Lett.* **13** 3531 (2013).
